# Selection of reference genes for quantitative gene expression normalization in flax (*Linum usitatissimum *L.)

**DOI:** 10.1186/1471-2229-10-71

**Published:** 2010-04-19

**Authors:** Rudy Huis, Simon Hawkins, Godfrey Neutelings

**Affiliations:** 1 UMR INRA-USTL 1281 Stress Abiotiques et Différenciation des Végétaux Cultivés, Bât. SN2, Université Lille 1, F-59655 Villeneuve d'Ascq, France

## Abstract

**Background:**

Quantitative real-time PCR (qRT-PCR) is currently the most accurate method for detecting differential gene expression. Such an approach depends on the identification of uniformly expressed 'housekeeping genes' (HKGs). Extensive transcriptomic data mining and experimental validation in different model plants have shown that the reliability of these endogenous controls can be influenced by the plant species, growth conditions and organs/tissues examined. It is therefore important to identify the best reference genes to use in each biological system before using qRT-PCR to investigate differential gene expression. In this paper we evaluate different candidate HKGs for developmental transcriptomic studies in the economically-important flax fiber- and oil-crop (*Linum usitatissimum *L).

**Results:**

Specific primers were designed in order to quantify the expression levels of 20 different potential housekeeping genes in flax roots, internal- and external-stem tissues, leaves and flowers at different developmental stages. After calculations of PCR efficiencies, 13 HKGs were retained and their expression stabilities evaluated by the computer algorithms geNorm and NormFinder. According to geNorm, 2 Transcriptional Elongation Factors (TEFs) and 1 Ubiquitin gene are necessary for normalizing gene expression when all studied samples are considered. However, only 2 TEFs are required for normalizing expression in stem tissues. In contrast, NormFinder identified glyceraldehyde-3-phosphate dehydrogenase (GADPH) as the most stably expressed gene when all samples were grouped together, as well as when samples were classed into different sub-groups.

qRT-PCR was then used to investigate the relative expression levels of two splice variants of the flax *LuMYB1 *gene (homologue of *AtMYB59*). *LuMYB1-1 *and *LuMYB1-2 *were highly expressed in the internal stem tissues as compared to outer stem tissues and other samples. This result was confirmed with both geNorm-designated- and NormFinder-designated-reference genes.

**Conclusions:**

The use of 2 different statistical algorithms results in the identification of different combinations of flax HKGs for expression data normalization. Despite such differences, the use of geNorm-designated- and NormFinder-designated-reference genes enabled us to accurately compare the expression levels of a flax MYB gene in different organs and tissues. Our identification and validation of suitable flax HKGs will facilitate future developmental transcriptomic studies in this economically-important plant.

## Background

Flax is one of the earliest cultivated plants known to man and the remains of seeds found in different archeological sites suggest that it was also used before domestication, most probably for textiles [1]. Cultivated flax (*Linum usitatissimum *L.) is an annual, diploid, self-pollinated crop used as a source of both high-quality, cellulose-rich bast fibers and oil. The main areas of fiber production are currently found in France (77,000 ha) with an annual turnover of over 200 million euros followed by Russia and China (80,000 ha). In contrast, oil varieties (linseed) are mainly grown in India (930,000 ha), Canada (811,000 ha) and China (570,000 ha) [2].

Although flax fibers have now been largely replaced in textiles by cotton or synthetic fibers, they still are used in the fabrication of high-quality linen material and are also incorporated in the polymeric matrix during the fabrication of biocomposites in order to improve their mechanical properties [3].

In the flax stem, fibers are derived from the procambial cells of the protophloem [4] and are referred to as bast fibers. Individual bast fibers are extremely-long (≤ 77 mm) single cells [5] characterized by the presence of a very thick secondary cell-wall containing high amounts of cellulose and very low amounts of lignins as compared to the shorter fibers found in the xylem [6]. Bast fibers provide mechanical support to the plant and are organized in bundles that occupy the great majority of the external tissues between the epidermis and the vascular cambium. Flax fiber extraction is initiated during the retting process when stems are left on the ground in order to promote microbiological-mediated separation of the fiber bundles from the surrounding tissues [7].

Secondary cell-wall formation is most often studied in woody species such as poplar, pine and eucalyptus, or else in model species such as *Arabidopsis *and tobacco. More recently, growing interest in the unusual structure of the secondary cell wall of bast fibers (high crystalline cellulose, low lignin) is stimulating transcriptomic and genomic studies in flax and other bast fiber species [8-10]. A better understanding of the molecular regulation controlling fiber cell-wall metabolism should increase our knowledge of the very complex coordination necessary for the production and assembly of different polymers within the cell wall.

Flax is a suitable model for genetic and functional genomics as it is autogamous, easy to grow, has a relatively short vegetative stage, and can be genetically transformed [11-13]. The first studies on flax DNA structure were undertaken almost thirty years ago [14,15] and the first cDNA sequence was deposited in Genbank in 1993 [16]. Previous analyses of the flax transcriptome have mainly involved studies of single gene expression by Northern blot [17-23] or multiple gene expression by classical RT-PCR [24]. Only relatively few studies [10,25] have reported the use of qRT-PCR in flax. Quantitative real-time RT-PCR (qRT-PCR) is currently the most accurate method for detecting low abundant mRNAs and is capable of detecting slight variations in gene expression in different tissues of the same plant [26]. When compared to traditional methods used to evaluate transcript accumulation, the main advantages of qRT-PCR are its very high sensitivity and specificity. However, in order to obtain reliable and reproducible results, it is necessary to include suitable internal controls. Indeed, the high sensitivity of qRT-PCR can lead to misinterpretation of expression data [27], especially when biological samples show similar gene expression levels. Slight variations in the RNA integrity or quantity, in the reverse transcription yield, or more generally in the transcriptional activity of the studied organ can all have a major impact on quantification. It is therefore absolutely necessary to develop a normalisation strategy before undertaking gene expression studies. The identification of suitable control genes is thus an important challenge in transcriptome analyses and the major aim is to identify genes ubiquitously expressed in every tissue independently of the experimental context [28].

In the past a number of different genes were commonly used for normalising expression, especially in classical RT-PCR approaches. Most of these genes proved to be suitable for such studies at the time, mainly because of the low sensibility of RT-PCR. More recently, the same reference genes have also been used for qRT-PCR and it has been assumed that their expression is perfectly stable even though this has not necessarily been experimentally verified. Examples of such commonly-used genes include GAPDH (glyceraldehyde-3-phosphate dehydrogenase) [29], cyclophilin and 18S rRNA [30], actin [31], EF1alpha [32], ubiquitin [33] and beta tubulin [34].

Surprisingly, there are relatively few reports concerning the study of stably-expressed genes, referred to as housekeeping genes (HKGs), in different plant species, even though qRT-PCR is routinely used in many research projects. Suitable HKGs have been identified in model species with sequenced genomes such as rice [35], *Arabidopsis *[36], grapevine [37] and poplar [38], as well as in economically-important crops such as wheat [39], barley [40], Coffee [41], tomato [42], potato [43] and soybean [44]. In most cases, gene expression stability was determined by using statistical approaches such as geNorm [45] and NormFinder [46]. The geNorm algorithm allows the identification of the most suitable reference gene(s) and the optimal number of genes that should be used. It relies on a pairwise comparison and evaluates the variation of relative quantity ratios for each gene pair in a set of expression data. The NormFinder algorithm depends on a statistical and mathematical model that not only estimates the overall expression variation of a candidate gene, but also considers the variation between the chosen subgroups.

In the present study, we have evaluated the expression profiles of 13 putative HKGs during the development of flax plants with a special focus on the stem which contains two major cell-wall tissue types: 1) inner tissues (lignin-rich secondary cell walls of xylem) and 2) outer tissues (lignin-poor, cellulose-rich secondary cell walls of bast fibers). The two algorithms geNorm and NormFinder were used in order to determine the best reference genes needed for normalisation. The selected HKGs were then used to normalise gene expression in flax for an investigation of the expression of *LuMYB1 *- a flax MYB transcription factor highly similar to *AtMYB59 *[47].

MYB transcription factors are of particular interest in studies on secondary cell wall development since they have been shown to regulate the expression of genes coding enzymes of the phenylpropanoid biosynthetic pathway responsible for the production of lignin monomers [48,49]. We decided to investigate the expression profiles of *LuMYB1 *since initial transcriptome studies in our laboratory (data not shown) had indicated that the gene was most highly expressed in inner stem tissues and therefore potentially associated with secondary cell wall formation in flax xylem tissues. In addition, *AtMYB59 *has been shown to undergo alternative splicing leading to different transcripts in rice and *Arabidopsis*, and therefore represents an interesting model for gene regulation studies.

## Results

### Selection of candidate reference genes and amplification specificity

Many reference genes, assumed to have a stable expression have been used to normalize expression in transcriptomics. In this study, primers were designed for 20 commonly used housekeeping genes (HKGs) representing different functional classes in flax. Sequences were identified after performing BLASTX searches on a collection of ESTs obtained from a flax fiber-rich cDNA library [24] and at the NCBI for the tubulin sequence. Finally, 13 primer pairs were selected on the basis of their amplification efficiency. The candidate reference genes including those encoding actin, cyclophilin, two elongation factors, five different eukaryotic translation initiation factors, GAPDH, ubiquitin, ubiquitin extension protein and tubulin are described in Table 1. The expression stabilities of these potential flax HKGs were assessed by qRT-PCR on a series of two biological repeats of 13 tissue samples obtained from different tissues at 3 developmental stages. Theses samples correspond to: 1) leaves, apical- and medium-stem tissues (vegetative stage), 2) roots, flowers, apical inner- and outer-stem tissues, medium inner- and outer-stem tissues (flowering stage) and 3) apical inner- and outer-stem tissues, medium inner- and outer-stem tissues (green-capsule stage). The different stem samples and developmental stages were chosen since previous studies [6,50] have shown that the main developmental modifications associated with bast fiber formation and cell wall maturation occur in the apical and medium stem tissues between the vegetative and green capsule stages.

**Table 1:** Flax candidate HKG description and comparison with *Arabidopsis *orthologs.

Gene abbreviation	GenBank accession number	Gene description	*Arabidopsis *ortholog locus	*Arabidopsis *BlastX E-value
ACT	GR508908	Actin	NM_121018	1e-145

CYC	GR508910	Cyclophilin	AF020433	1e-48

EF1A	GR508911	Elongation Factor 1-α	AK230352	2e-155

EF2	GR508907	Elongation Factor 2	AC009894	3e-162

ETIF1	GR508906	Eukaryotic translation initiation Factor 1	NM_122959	2e-49

ETIF3E	GR420420	Eukaryotic translation initiation Factor 3 E	NM_115589	2e-54

ETIF3H	GR420421	Eukaryotic translation initiation Factor 3 H	NM_100960	1e-115

ETIF4F	GR420423	Eukaryotic translation initiation Factor 4 E	ATU62044	8e-72

ETIF5A	GR508912	Eukaryotic translation initiation Factor 5 A	NM_101261	4e-78

GAPDH	CV478202	Glyceraldehyde 3-phosphate dehydrogenase	NM_101214	1e-120

UBI	CV478829	Ubiquitin	DQ793133	3e-71

UBI2	GR508909	Ubiquitin extension protein	NM_130278	5e-66

TUA	CA482867	α-Tubulin	AK226954	8e-103

**Figure 1:**
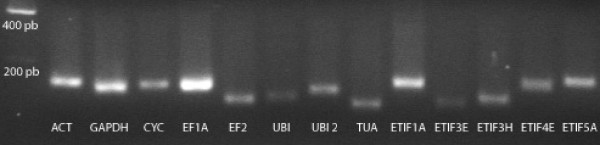
**Real-time quantitative RT-PCR amplification specificity**. Amplified fragments obtained after qRT-PCR were separated by agarose gel electrophoresis. Amplification primers were designed for Actin (*ACT*), *GAPDH*, Cyclophilin (*CYC*), Elongation factor 1A (*EF1A*) and 2 (*EF2*), Ubiquitin (*UBI*), Ubiquitin extension protein (*UBI 2*), Tubulin (*TUA*), Eucaryotic Translation Initiation Factors 1A (*ETIF1A*), 3E (*ETIF3E*), 3H (*ETIF3H*), 4E (*ETIF4E*) and 5A (*ETIF5A*).

Specific transcript amplification was confirmed by the presence of a single peak in the melting curve obtained after amplification. The amplified products were also further analyzed by agarose gel electrophoresis and ethidium bromide staining (Figure 1). Only a single band with the expected size was detected in each experiment. The amplification efficiencies are indicated in Table 2.

**Table 2:** Description of candidate reference genes, primers and amplicons for flax housekeeping gene selection.

Gene abbreviation	Gene description	Primer Sequence (Forward/Reverse Primer)	Amplicon length (bp)	Efficiencies (%)
ACT	Actin	5'-TCCAGGCCGTTCTTTCTCTA-3'	153	97.9
		5'-CTGTAAGGTCACGACCAGCA-3'		
CYC	Cyclophilin	5'-TGATTGCGGTCAGCTGTAAG-3'	147	93.5
		5'-AGGTGAAACGCTAGGCAGAA-3'		
EF1A	Elongation Factor 1-α	5'-GCTGCCAACTTCACATCTCA-3'	140	101.9
		5'-GATCGCCTGTCAATCTTGGT-3'		
EF2	Elongation Factor 2	5'-GTGGTGCTGAGATCACGAAA-3'	109	98.9
		5'-AGACGGTTATGCTTGTTGGG-3'		
ETIF1	Eukaryotic translation initiation Factor 1	5'-CCTTGTAGGGCTGAGGGATT-3'	145	104.8
		5'-CTCATCAAGACCACCAGCAA-3'		
ETIF3E	Eukaryotic translation initiation Factor 3 E	5'-TTACTGTCGCATCCATCAGC-3'	106	101.8
		5'-GGAGTTGCGGATGAGGTTTA-3'		
ETIF3H	Eukaryotic translation initiation Factor 3 H	5'-CAGCGTGCTTGAAGTAACCA-3'	115	101.7
		5'-AACCTCCCTCAAGCATCTCA-3'		
ETIF4E	Eukaryotic translation initiation Factor 4 E	5'-TGTGGCTCGAAACTCTGATG-3'	145	103.5
		5'-CCCATCTGGACAGCTTCATT-3'		
ETIF5A	Eukaryotic translation initiation Factor 5 A	5'-TGCCACATGTGAACCGTACT-3'	159	103.1
		5'-CTTTACCCTCAGCAAATCCG-3'		
GAPDH	Glyceraldehyde 3-phosphate dehydrogenase	5'-AGGTTCTTCCCGCTCTCAAT-3'	138	101.7
		5'-CCTCCTTGATAGCAGCCTTG-3'		
UBI	Ubiquitin	5'-CTCCGTGGAGGTATGCAGAT-3'	114	94.7
		5'-TTCCTTGTCCTGGATCTTCG-3'		
UBI2	Ubiquitin extension protein	5'-CCAAGATCCAGGACAAGGAA-3'	129	98.6
		5'-GAACCAGGTGGAGAGTCGAT-3'		
TUA	α-Tubulin	5'-CCTGTTGGGAGCTTTACTGC-3'	100	102.6
		5'-AAGGTGTTGAAGGCATCGTC-3'		

### Expression profile of the reference genes

**Figure 2:**
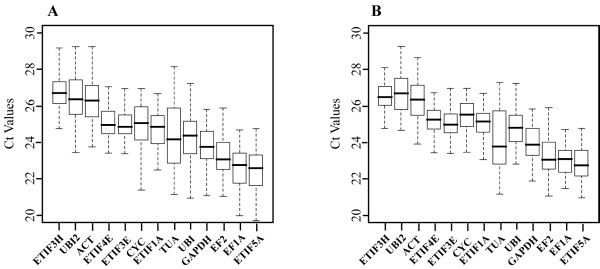
**Expression levels of candidate reference genes in flax tissue samples**. Box-plot graphs of Ct values for each reference gene in 13 flax tissue samples (A) or only in stem tissues (B). The black lines show the median values surrounded by lower and upper boxes indicating the first and third quartile. The vertical lines (whiskers) indicate value ranges. The gene abbreviations are the same as in Figure 1.

In order to determine the expression rates of the selected genes, all PCR assays were performed in triplicate. The Ct values for the 13 genes were pooled so that the expression levels of each gene could be compared. These values ranged from 19.70 to 29.25 in all tested samples (Figure 2A) and from 20.93 to 29.24 if only stem tissues were considered (Figure 2B). Six genes (*TUA*, *UBI*, *GAPDH*, *EF2*, *EF1A*, and *ETIF5A*) showed the highest expression levels irrespective of whether all tissues or just stem tissues are considered. *ETIF5A *showed the lowest median and mean Ct values and *UBI2 *showed the highest values in both total and stem tissues.

With regards to gene expression variation, *TUA *showed the highest value (6.98 cycles) and *ETIF4E *showed the lowest (3.67 cycles) when all tissues are considered. As might be expected, expression variation was lower when only stem samples are considered. Once again *TUA *showed the highest value (6.1 cycles) whereas the lowest value was observed for *EF1A *(3.2 cycles). These results indicated that none of the selected genes showed a near constant expression level and it was therefore necessary to evaluate the best candidates for gene expression normalization.

### Expression stability analysis

Since the 13 candidate HKGs show wide variations in expression levels in different flax tissues, it is necessary to use statistical approaches to rank the expression levels and determine the number of housekeeping genes necessary for accurate gene-expression profiling in the selected tissues. We decided to use the two most widely-used algorithms, geNorm and NormFinder.

**Figure 3:**
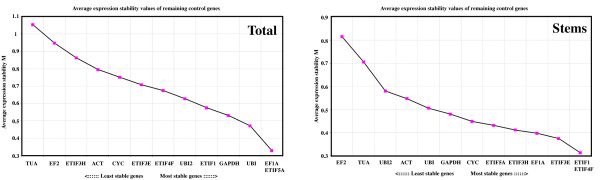
**Average expression stability values (M) of flax candidate reference genes**. M values of the reference genes were calculated with the geNorm algorithm. Ranking of the stability was performed on 13 different flax tissues (A) or on stem tissues (B). The lowest average expression stability value indicates the most stably expressed gene. The gene abbreviations are the same as in Figure 1.

The geNorm program can be used to identify (in a given set of candidate genes) those genes that are the most stably expressed in different organs or tissues [45]. In this program, an average expression stability value M is calculated for all candidate genes and those genes with the lowest M values are considered to be the most stable. We used geNorm to analyse candidate gene expression levels in 1) total tissues and 2) stem tissues only (Figure 3). When all samples were analysed together, the M value was lowest for *EF1A *and *ETIF5A *indicating that they are the most stably expressed gene pair out of the 13 candidates. The alpha-Tubulin gene was the least stably expressed. When only stem tissues were considered, *ETIF1 *and *ETIF4F *proved to be the best candidates for normalization (low M value) while *EF2 *was the worst (high M value). However, in all cases the M values were less than 1 indicating that the selected genes all show relatively acceptable expression stabilities. Nevertheless, our results highlight the fact that it is probably better to choose different reference genes depending on the biological samples to be studied. For example, when only flax stem tissues are considered, the five Eukaryotic translation initiation factors are found amongst the six most stably-expressed genes. In contrast, when all tissues are considered the same five genes show less overall stability and *ETIF3H*, for example, is the third least-stable gene. Similarly, some genes are stably-expressed when all tissues are considered but appear to be less stable when only stem tissues are taken into account. For example, the ubiquitin gene is the third most-stably expressed gene in total tissues but is only the eighth most stably expressed gene in stems tissues. Finally, the alpha tubulin and elongation factor 2 genes are the two least stable genes in both total tissues and in stem tissues.

**Figure 4:**
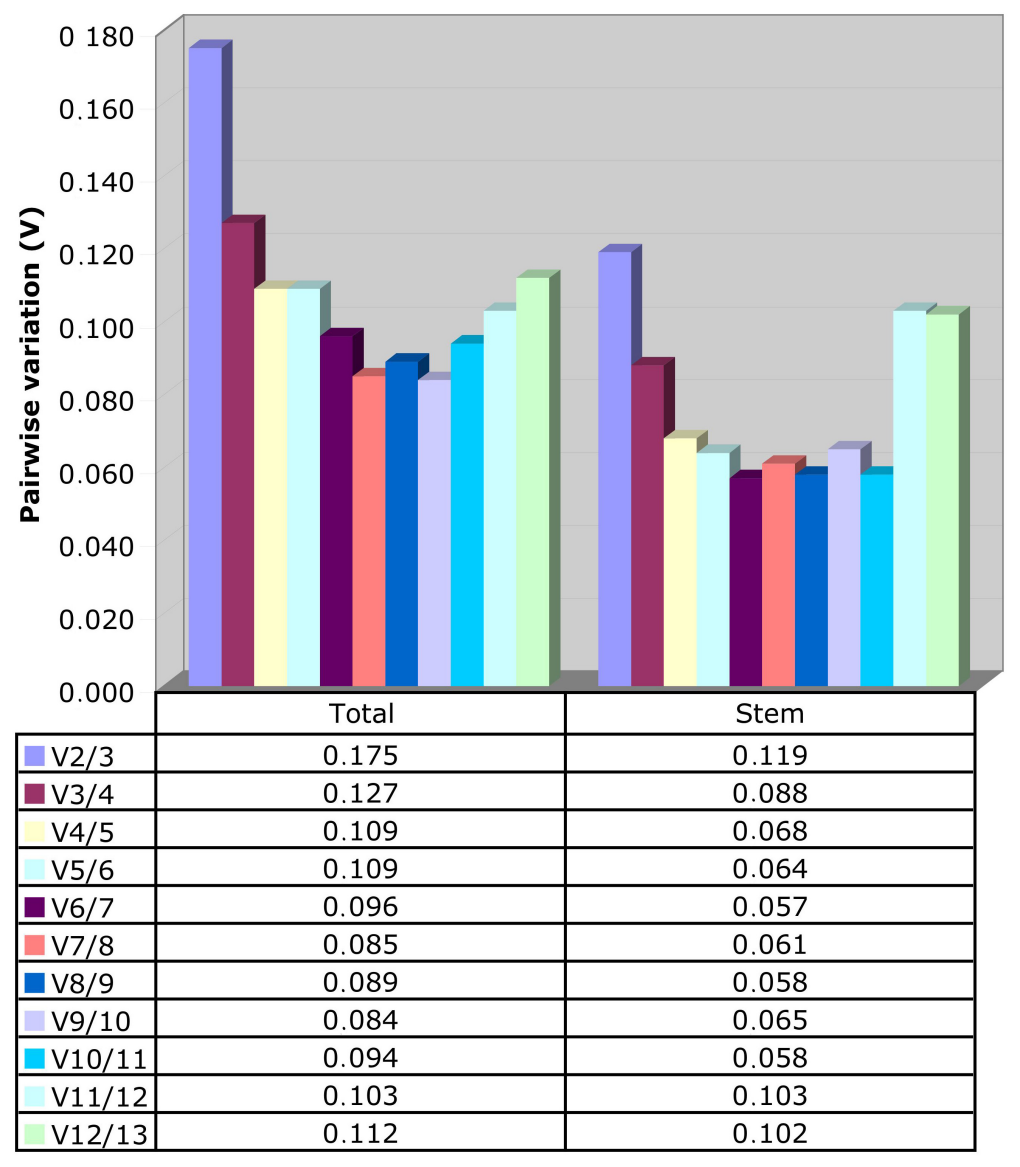
**Determination of the optimal number of reference genes**. Pairwise variation (V) values were calculated with the geNorm algorithm. Values under 0.15 indicate that no additional genes are required for the normalization of expression in the selected tissues.

We also used the geNorm program to calculate the optimal number of HKGs required for accurate normalization in flax (Figure 4). The software calculates the pairwise variation V_n_/V_n+1 _between two sequential normalization factors NF_n _and NF_n+1 _in order to determine the necessity of including an additional control gene for normalization. As long as the V value remains higher than 0.15, an additional reference gene should be added. Our results (Figure 4) showed that the pairwise variation values changed depending upon the samples analyzed. For example, in all tissues, normalization requires the use of three reference genes since only the V3/4 value (0.127) is less than 0.15. However, gene normalization in stem tissues requires the use of only two reference genes since the V2/3 value (0.119) is inferior to the 0.15 cut-off level.

We then used the NormFinder algorithm [46] to identify the most stable gene among our candidates and test whether the use of only one HKG would be suitable for gene expression studies. The NormFinder algorithm requires the analysis of a minimum of 3 genes and a minimum of 2 samples per group. It can analyze the expression data obtained from quantitative methods and choose the best normalization gene from a set of candidates. The software calculates and ranks the stability values for candidate genes within the sample set investigated according to their expression profile. The lowest stability value indicates the most stably expressed gene.

The results of our NormFinder analysis in flax are shown in Table 3. The samples were assembled in one main group or several sub-groups. If all the samples were combined in one group, the best reference gene was *GAPDH *(stability value 0.069). This gene was also detected as the most stably expressed gene (stability values 0.04 - 0.061) when the samples were distributed into either 2 or 3 sub-groups. However, if the analysis was performed on one main group containing only stem tissues, the most stable gene proved to be *ETIF3H *(stability value 0.056). Nevertheless, in this case *GAPDH *proved to be the second-most stably expressed gene (stability value 0.064).

**Table 3:** Ranking of candidate genes according to NormFinder.

	1 Group				2 Groups		3 Groups			
	**Total**		**Stems**		**Stems/Others**		**Apical/Medium/Others**		**Ext/Int/Others**	
**Rank**	**Gene**	**Stability**	**Gene**	**Stability**	**Gene**	**Stability**	**Gene**	**Stability**	**Gene**	**Stability**

**1**	GAPDH	0,069	ETIF3H	0,056	GAPDH	0,061	GAPDH	0,048	GAPDH	0,040
**2**	UBI2	0,099	GAPDH	0,064	ETIF4F	0,072	UBI2	0,071	UBI2	0,066
**3**	UBI	0,103	ETIF3E	0,080	UBI2	0,081	ETIF3E	0,079	TUA	0,070
**4**	ETIF3E	0,108	ETIF5A	0,080	ETIF3E	0,082	ETIF4F	0,081	UBI	0,075
**5**	EF1A	0,112	EF1A	0,087	UBI	0,104	ACT	0,091	ACT	0,076
**6**	ETIF5A	0,118	CYC	0,100	ACT	0,106	UBI	0,094	ETIF5A	0,076
**7**	ETIF4F	0,138	EF2	0,103	EF1A	0,129	CYC	0,113	EF1A	0,076
**8**	CYC	0,145	UBI	0,105	CYC	0,130	EF1A	0,113	ETIF1	0,078
**9**	EF2	0,145	ACT	0,107	ETIF5A	0,135	ETIF5A	0,118	ETIF4F	0,082
**10**	ETIF1	0,157	ETIF1	0,131	EF2	0,142	EF2	0,126	ETIF3E	0,084
**11**	ACT	0,174	UBI2	0,133	ETIF1	0,149	ETIF1	0,127	CYC	0,095
**12**	ETIF3H	0,189	ETIF4F	0,137	TUA	0,180	TUA	0,166	EF2	0,100
**13**	TUA	0,206	TUA	0,176	ETIF3H	0,208	ETIF3H	0,182	ETIF3H	0,124

**Best combination of two genes**					EF1A EF2	0.028	EF1A ETIF5A	0,03	ETIF3E UBI	0,03

### *LuMYB1 *expression

**Figure 5:**
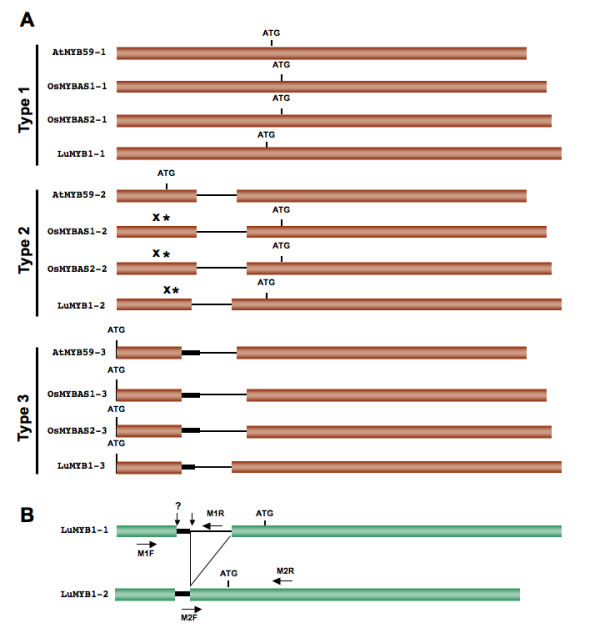
**Schematic representation of the differently spliced transcripts of MYB homologues**. A: The transcribed sequences are shown in red boxes while the spliced fragments are represented as thin lines. B: Representation of *LuMYB1 *splice variants. The spliced positions are indicated with vertical arrows. The primer positions used for qRT-PCR are shown with horizontal arrows. X indicates conserved but non-functional start codons and the asterisks indicate stop codons. ATG indicates functional start codons. The hypothetical *LuMYB1-3 *transcript should be formed by splicing at the position indicated by ?.

The use of the geNorm and NormFinder algorithms allowed the identification of different potential HKGs for normalization of gene expression levels in flax. In order to see whether the normalization by these different HKGs modified the qRT-PCR-determined expression levels of a gene of interest, we decided to analyze the developmental expression of a flax *MYB *gene, *LuMYB1*. We had previously isolated two different *LuMYB1 *transcripts, *LuMYB1-1 *and *LuMYB1-2 *during a flax EST sequencing project [24]. The alignment of the two sequences showed that they are perfectly identical except for the upstream region of *LuMYB1-2 *that lacks a 68 bp fragment. *LuMYB1 *is an ortholog of *AtMYB59 *which is found to undergo alternative splicing in *Arabidopsis *[47]. *AtMYB59 *gives rise to four spliced transcripts (Figure 5A), three of which encode proteins with either one or two MYB domains. A similar pattern of alternative splicing also occurs in two homologous genes (*OsMYBAS1*, *OsMYBAS2*) in rice. In this species, three of the four transcripts have been detected and can be aligned with *AtMYB59 *(Figure 5A). In flax, the *LuMYB1-1 *and *LuMYB1-2 *transcripts can also be aligned with the *Arabidopsis *and rice type 1 and 2 transcripts.

Interestingly, both the rice and flax (but not *Arabidopsis*) type 2 transcripts contain a stop codon close to (and in frame with) the corresponding ATG start codon in *Arabidopsis*. As a result in both rice and flax, translation is initiated at the next ATG that corresponds to the functional ATG in type 1 transcripts. In consequence, the flax type 1 and 2 ORFs, as in rice, are identical. We have not yet detected a flax type 3 transcript (*LuMYB1-3*) in our ESTs even though sequence data predicts the existence of such a transcript. Nevertheless, we designed qRT-PCR primers to specifically amplify the two longest flax transcripts. *LuMYB1-1 *was detected with the M1R primer located in the intron, and *LuMYB1-2 *with the M2F primer overlapping the 3' splicing site (Figure 5B).

**Figure 6:**
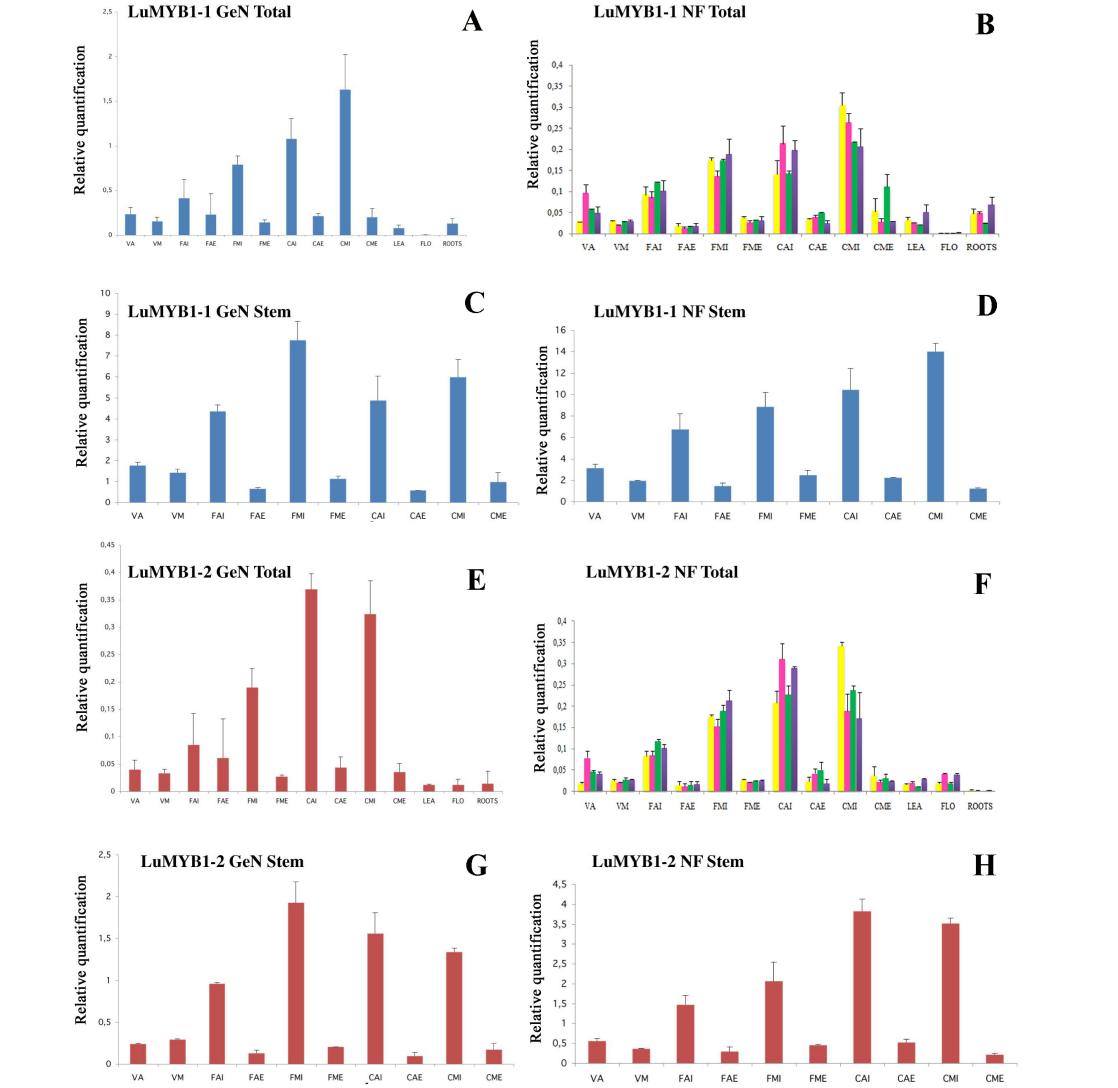
**Relative quantification of two *LuMYB1 *splice forms**. The relative expression levels of *LuMYB1-1 *(A, B, C and D) and *LuMYB1-2 *(E, F, G and H) were determined after normalization. The total tissue (A and E) or stem (C and G) sample sets were normalized by geNorm with *EF1A*, *ETIF5A *and *UBI *or with *ETIF1 *and *ETIF4F *respectively. The NormFinder algorithm was also used with different combinations of HKG. The total tissues (B and F) were divided in 1, 2 or 3 groups and normalized with *GAPDH *alone (1 group, yellow bar), *EF1A *and *EF2 *(stem/others, pink bar), *EF1A *and *ETIF5A *(apical/medium/others, green bar) *ETIF3E *and *UBI *(external/internal/others, blue bar). When the expression was measured only in one group containing the stem tissues (D and H), *ETIF3H *was used for normalization. V: vegetative F: flowering and C: green capsule stages; A: apical and B: basal parts of the stem; I: internal and E: external stem tissues; LEA: leaves; FLO: flowers. Values = relative expression levels +/- SD.

The relative expression levels of *LuMYB1 *were estimated in the same 13 flax samples that were used previously for the normalization studies (Figure 6). In order to see whether the use of different HKGs (chosen according to geNorm or NormFinder) modified the determined expression profiles of the 2 *LuMYB1 *splice variants, we compared different HKGs combinations in 1) all flax tissues and 2) in just stem tissues (Figure 6). For all tissues, normalization was accomplished by using the three most stable genes *EF1A*, *ETIF5A *and *UBI *(determined by the geNorm analysis), or by *GAPDH*, the most stable gene determined by NormFinder when all the samples were assembled into one main group (Table 3, Figure 6A, B). When only stem tissues were analyzed, normalization was accomplished by using the 2 most stable genes *ETIF1 *and *ETIF4F *(determined by geNorm), or *ETIF3H*, the most stable gene determined by NormFinder when only stem tissues are considered (Table 3, Figure 6C, D). Our results indicate that *LuMYB1-1 *is expressed in all tissues except for the flowers, and that the highest expression levels are found in inner stem tissues, regardless of whether HKGs identified by geNorm or NormFinder were used for normalization. However, since only 1 HKG (*GAPDH *or *ETIF3H*) is identified for all samples in the NormFinder algorithm when no sub-groups are defined, we decided to define different sub-groups so as to identify the best HKG couples for normalizing expression data. Different sub-groups were identified (Table 3, Figure 6B) in order to see whether this influenced the choice of HKG couples and subsequent expression normalization in different samples. When all samples were divided into 2 sub-groups (stems/other samples), NormFinder identified EF1A and EF2 as the most stable couple (stability value 0.028). When 3 sub-groups were identified, the best HKG couple identified by NormFinder depended upon the sub-groups chosen. For example, EF1A and ETIF5A (stability value 0.033) represented the best HKG couple for the sub-groups 'apical/medium/other samples', while ETIF3E and UBI (stability value 0.026) represented the best HKG couple for the sub-groups 'external/internal/other samples'. However, despite the fact that NormFinder identified different HKG couples depending upon the sub-groups defined, the use of these different HKG couples to normalize *LuMYB1-1 *expression data largely confirmed the expression profile previously identified by geNorm and NormFinder (no sub-group, 1 HKG). Determination of the *LuMYB1-2 *expression profile indicated that the gene was expressed in all tissues (including the flowers) and that, as previously observed for *LuMYB1-1*, the highest expression levels could be observed in the inner stem tissues (Figure 6E, F). This general expression pattern (highest expression in stem inner tissues) was obtained with both geNorm- and NormFinder-designated HKGs. As for *LuMYB1-1*, the definition of different sub-groups (and hence different HKG couples) did not fundamentally change the determined *LuMYB1-2 *expression profile. Nevertheless, our results underline the fact that the use of a particular algorithm and choice of sample set can influence the determined expression value of the gene of interest when the values are relatively close. For example, the use of geNorm for the determination of *LuMYB1-2 *expression in the stem group (Figure 6G) would suggest that there is little difference between expression levels in stem medium inner tissues at the flowering stage and stem apical inner tissues at the green capsule stage, whereas the use of other HKGs (Figure 6E, F, H) would suggest that *LuMYB1-2 *expression is higher in stem inner tissues at the green capsule stage.

## Discussion

Over the last years several very powerful techniques have been developed to detect differences in gene expression levels between different cell types, tissues and organs. Among these, qRT-PCR is now commonly used in many laboratories to undertake accurate expression profiling of different candidate genes that have been previously identified, either because they are known to be involved in a specific biological process, or else because they have shown an interesting expression profile in global expression analyses (microarrays).

However, reliable relative quantification can only be performed if an accurate normalization is performed following the choice and verification of suitable (i.e. stable) HKGs. Although microarray data have been analyzed to determine the most stably-expressed genes in *Arabidopsis *[36] and rice [51], it is not always possible to directly transpose suitable HKGs identified in one species to another species. For example *UBQ10 *is very stable in Arabidopsis [36] but not in rice [52], nor in soybean [44] nor in Brachypodium [53]. Therefore, since there are no universally-suitable reference genes, it is necessary to verify the expression levels of the candidate reference genes under the same experimental conditions as those used for the gene of interest.

In this study, we have measured the expression levels and stability of candidate genes in different tissues at three developmental stages in flax plants. Our major research interest in this species concerns the cell wall formation and development of the phloem (bast) fibers located in the external tissues of the stem. The polymer composition of the secondary cell walls in these long fiber cells (as well as that of the shorter xylem cells located in the inner tissues) has been previously analyzed [6] and we have shown that the abundance and structure of the phenolic polymer lignin varies according to the age and physiological state of the plant. In order to further investigate cell wall formation (including lignification) in flax, we are currently undertaking gene expression profiling by qRT-PCR on different tissues. However, due to important differences between the structure and the metabolic state of cells in flax inner and outer tissues, it is necessary to identify the most relevant combination of reference genes. Normalization of gene expression in flax has previously been done with either a homolog to an *Arabidopsis *NADH Ubiquinone oxidoreductase determined by cDNA-AFLP [25], or with an Elongation Factor *EF1A *homolog [10].

We originally selected 20 candidate HKGs from the literature and after determination of amplification efficiencies for the selected primers we retained 13 candidates. We then evaluated their normalization potential using 2 commonly-used algorithms (geNorm [45] and NormFinder [46]) in a systematic study of their expression stability in different flax tissues including stems, flowers, roots and leaves. The NormFinder algorithm [46] generally ranked GAPDH as the most stably expressed gene in all the flax samples irrespective of whether the samples were assembled into 1 main group or divided into several sub-groups. In only 1 case did NormFinder rank GAPDH as the second most stable gene. These results are consistent with those observed recently in *Brachypodium distachyon *[53] during cold/heat stress and in *Coffea arabica *[41] in different organs and tissues. In contrast to NormFinder, the geNorm algorithm indicated that we should use 3 HKGs (*EFIA*, *ETIF5A*, *Ubiquitin*) for studies involving a mix of root, leaf, stem and flower tissues. However, the same program indicated that only 2 genes (*ETIF1*, *ETIF4F*) should be used when just stem tissues were considered. While *EF1A *is often described as a stable gene and has been used as a reference in many species [37,43,52,54], there are only relatively few reports of Eucaryotic Translation Initiation Factor genes being used as reference genes. For example in soybean, *ETIF1A *and *ETIF1B *genes were ranked as the most stable genes tested when 21 samples were pooled, or when photoperiodic treatments were modified [44]. In contrast, expression patterns of several translation initiation factors were shown to be unstable in wheat [55], and have been excluded as good candidate reference genes in poplar [38] and Darnel ryegrass [54].

We also evaluated the stability of tubulin and actin genes that are very often used to normalize expression data. Although actin has been shown to be a suitable normalization gene in developmental studies [44], it also appears to be unstable in many biological processes [27]. In the same way, tubulin has been shown to be stable during development in orobranche [56], but is apparently unstable (geNorm analyses) during development [53] and abiotic stress [54]. Our results would also suggest that these 2 genes are among the most instable for expression profiling during development in flax.

Altogether, our results showed that the geNorm and NormFinder algorithms came to different conclusions concerning the best candidate HKG(s) to use for expression normalization in flax. In order to see whether the use of these different HKGs modified the determined expression profiles (and hence possible interpretations of biological role) of a gene of interest, we analyzed the expression of *LuMYB1 *in different tissues and organs of Flax. *LuMYB1 *is a homolog of *AtMYB59*, a gene that has been shown to undergo alternative splicing in *Arabidopsis *and rice [47]. We decided to investigate the expression profile of *LuMYB1 *since our team is interested in gaining a better understanding of the regulation of cell wall formation in flax and different MYB transcription factors have been previously shown to play an important role in controlling this process [48]. In addition, preliminary transcriptome data in our lab had indicated that *LuMYB1 *was highly expressed in stem inner tissues. Such an observation could suggest that this gene is involved in the transcriptional regulation of cell-wall genes during the formation of xylem.

Our results showed that the 2 different flax spliceforms (*LuMYB1-1 *and *LuMYB1-2*) are strongly expressed in the inner stem tissues, but only expressed at low levels in external stem tissues, leaves and roots. In addition, only *LuMYB1-2 *is expressed in flowers. Such a result is interesting since previous work [47] has shown that the corresponding *Arabidopsis *gene is mainly expressed in roots, leaves and seedlings, but only poorly expressed in stem tissues. More recently [57], *AtMYB59 *has been shown to be specifically expressed during the S phase of the cell cycle in *Arabidopsis *cell suspensions, suggesting that this gene plays a key role in cell cycle regulation. Our observation that the corresponding flax gene (both spliceforms) are highly expressed in inner stem tissues could also suggest a role for *LuMYB1 *in cell cycle events since these flax tissues are the site of intense mitotic activity associated with the division of vascular cambium initials to form new xylem cells. However, further work is obviously necessary to confirm this hypothesis.

In addition, our results also revealed that both the choice of HKGs and the decision or not to analyze samples together or in sub-groups modified the determined expression profiles for the gene of interest *LuMYB1*. Although such choices did not affect the identity of the 4 (stem) tissues showing the highest expression levels, they did affect the ranking of these different tissues. Such an observation suggests that care should be taken when interpreting the biological significance of small differences in gene expression, and that in this case, the researchers should probably consider using different normalizations to verify their data. In addition, it is clear that functional approaches are also necessary to fully understand the role of a given gene in a particular biological process. Nevertheless, our results showing important differences in *LuMYB1 *expression levels (regardless of the normalization type) would suggest that this gene is potentially associated with the formation of xylem tissue in flax stems.

## Conclusions

A considerable quantity of transcriptional data is currently available for major model plant species and *in silico *analyses can therefore be used to identify suitable HKGs for gene expression normalization in these species. However, for most other plant species, the suitability of potential HKGs identified in the literature or in heterologous databases must be verified by qRT-PCR. In this study, we have identified several suitable reference genes for studying developmental gene expression in flax, with a special focus on stem tissues. The use of HKGs identified by both geNorm and NormFinder algorithms allowed us to determine the expression profiles of a gene of interest (*LuMYB1*) in a large range of different flax tissues. Our results also showed that certain classically-used reference genes such as actin and tubulin are not necessarily the most suitable for studies of quantitative gene expression in flax. In conclusion, our study has identified suitable HKGs for future developmental transcriptome studies in flax. These genes also represent potentially interesting targets for normalizing gene expression in flax under stress conditions, although it would obviously be necessary to verify their stabilities in this case.

## Methods

### Plant material

Flax plants (*Linum usitatissinum *L. cv Barbara were grown in a greenhouse under 16 h/20°C day and 8 h/18°C night conditions. The plants were harvested at three developmental stages: (1) vegetative (56 days after sowing), (2) flowering (131 days after sowing, 50% open flowers) and (3) green capsule stage (159 days after sowing). Following harvest, organs were dissected and the stems were divided into three equal parts: - apical, medium and basal (the latter was discarded because the secondary cell walls of bast fibers have obtained their maximum thickness in this part of the stem and residual metabolism is low and unrelated to fiber maturation events).

For the flowering and green capsule stages, the outer fiber-bearing tissues were separated from the inner woody tissues. The tissues were immediately frozen in liquid nitrogen and stored at -80°C.

### RNA isolation, quality control and cDNA synthesis

Frozen tissues were ground in liquid nitrogen using a mortar and a pestle. Total RNA was extracted using the RNeasy Plant Mini Kit (Qiagen) according to the manufacturer's instructions. RNA purity was assessed on a biophotometer (Eppendorf) by determining the OD260/OD280 and OD260/OD230 ratios, which were between 1.8 and 2. Potentially-contaminating DNA was eliminated by treatment with DNAse I using the DNA-free kit (Ambion). RNA concentration and quality were determined by capillary electrophoresis on an Experion labchip electrophoresis system (Bio-Rad) One microgram of total RNA was reverse-transcribed using the Iscript cDNA synthesis kit (Bio-Rad) according to the manufacturer's instructions. The cDNAs were diluted 1:256 with nuclease free water.

### Design and validation of reference gene primers

Specific primer pairs were first designed for 20 commonly used housekeeping genes representing distinct functional classes and gene families. These include ubiquitins, actin, tubulin, elongation factors, cytosolic cylophilins and translational initiation factors (Table 2). The sequence of these genes were identified by BLAST searches (Table 1) in a flax EST database obtained from a cDNA library derived from the outer fiber-bearing tissues of flax [24]. For primer design, Primer3 software [58] was used (160 bp maximum length, optimal Tm at 60°C, GC % between 20% and 80%). The absence of secondary structure in the primer annealing fragments was verified using mfold [59]. Five-point standard curves of a 4-fold dilution series (1:16 to 1:4096) were used to calculate the PCR efficiency (E) of each primer pair. The PCR efficiency is given by the equation E = (10^(-1/m) ^-1) × 100 where m is the slope of the linear regression model fitted over log transformed data of the input cDNA concentration versus Ct values according to the linear equation y = m log(x) + b. After evaluation of the 20 primer pairs, 13 were retained on the basis of their amplification efficiency value (93-105%).

### qRT-PCR conditions and analyses

The qRT-PCRs were carried out in 96-wells plates with a MyIQ real time PCR detection system (Bio-Rad) using Quantitect SYBR Green PCR Kit (Qiagen) in a reaction volume of 20 μL (5 μL diluted cDNAs, 10 μL of 2× SYBR Green mix and primer pairs at 0.4 μM). Aliquots from the same cDNA solutions were used with all primer sets in each experiment. All PCR reactions were performed under the following conditions: 95°C for 15 min, 40 cycles of 10 s at 95 °C and 30 s at 60°C. For each primer pair, a melting curve was generated in order to confirm the specificity of the amplification and the PCR products were checked on a 4% agarose gel. Data were analysed using Bio-Rad iQ5 software. The efficiency (E) value of each reaction was between 1.93 and 2.04 with R^2 ^values higher than 0.998. Each experiment was repeated three times on two biological replicates, each one represented by three technical repetitions. PCR reactions on samples lacking the cDNA template or the reverse transcriptase during the cDNA synthesis were also performed as negative controls for each primer pair.

### Statistical analyses of gene expression stability

The stability of the candidate reference genes was evaluated by two statistical approaches. In both approaches, expression levels were expressed relative to the sample with the highest expression. Ct values were converted into relative quantities and imported into the geNorm v3,5 software http://medgen.ugent.be/~jvdesomp/genorm/ and into the NormFinder software http://www.mdl.dk/publicationsnormfinder.htm.

The geNorm algorithm first calculates an expression stability value (M) for each gene and then the pairwise variation (V) of this gene with the others. All the tested genes are ranked according to their stability in the tested tissues and the number of HKGs necessary for an optimal normalization is indicated. NormFinder also ranks the stability of the tested genes, but independently of each other.

### Expression of *LuMYB1*

In order to validate the selected HKG, the relative expression level of *LuMYB1 *was calculated for each cDNA sample. Two primer pairs were designed in order to discriminate the two splice variants *LuMYB1-1 *[genbank:GQ374577] (M1F: GAGGACATCCTCCTGGTCAA; M1R: AGACCAACCTTCCCCAGATT) and *LuMYB1-2 *[genbank:GQ374578] (M2F: GGAGACGCGTAATAGGTTTG; M2R: GCGAGCAATTCTTGACCATCTG). For this study, two biological repeats of three technical measures were made in the same conditions as described above. The expression levels of the two forms were calculated according to the equation:

## Authors' contributions

RH performed the sample preparations, experimental procedures and data analysis. SH supervised the study and critically revised the manuscript. GN participated in the qPCR experiments, designed this work and wrote the manuscript. All authors read and approved the final manuscript.
